# The Effects of Climate Change and Greening of Vegetation on Spatiotemporal Variation of Evapotranspiration in the Haihe River Basin, China

**DOI:** 10.1002/ece3.71092

**Published:** 2025-03-06

**Authors:** Yang Chen, Se Chai, Wenjie Chen, Jiangzhou Xia

**Affiliations:** ^1^ Tianjin Key Laboratory of Water Resources and Environment Tianjin Normal University Tianjin China

**Keywords:** climate change, evapotranspiration, greening of vegetation, Haihe River basin, satellite‐based model

## Abstract

Highly accurate evapotranspiration (ET) estimation and understanding the impacts of climatic and land use change on ET are essential for water resources management in the Haihe River Basin (HRB). This study estimated spatial and temporal changes of ET and its drivers over the period 2000–2020, using the Priestley‐Taylor Jet Propulsion Laboratory (PT‐JPL) model. Validation performed with the observations of 11 eddy covariance sites showed that the PT‐JPL model can simulate ET with high accuracy (*R* ^2^ = 0.64, RMSE = 1.32 mm/day, NSE = 0.57). During the 21‐year study period, the mean annual ET in HRB was 583 mm/year and showed an insignificant increasing trend (0.45 mm/year). Canopy transpiration (ET_c_, 2.96 mm/year) and interception evaporation (ET_i_, 0.74 mm/year) significantly increased whereas soil evaporation (ET_s_, −3.25 mm/year) significantly decreased. The mean annual net radiation (Rn), relative humidity (Rh), and wind speed (Ws) showed insignificant decreasing trends. In contrast, mean annual air temperature (Tm), vapor pressure deficit (VPD), and precipitation showed insignificant increasing trends. The significantly increased leaf area index (LAI) demonstrated that vegetation in the HRB is greening. We explored the relationship between ET and its components to climate and vegetation parameters. The results showed that net radiation was the most important parameter for ET variations. Vegetation and temperature had large impacts on ET_c_. Vegetation greening in HRB dominates the increasing trend in ET_c_. Net radiation and relative humidity showed an important role in changes in ET_s_. Temperature and vegetation were key impact parameters for ET_i_. The increase in ET_i_ is mainly located in the region of forests, which is due to the forest protection and afforestation projects in HRB. This study highlights the importance of isolating the contributions of vegetation and climate changes to the changes in ET and its components, which is useful for water resources management in HRB and other regions of the world.

## Graphical abstract

The Haihe river basin (HRB) experienced significant greening of plants, causing an increase in evapotranspiration (ET). Canopy transpiration and interception evaporation significantly increased. The monitoring of water consumption by greening of plants should be strengthened.

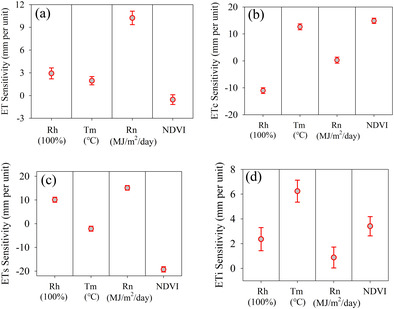

## 1 Introduction

The Haihe River Basin (HRB) is one of the seven major river basins in China (Li et al. 2018). The cities in the HRB include the economic, cultural, and political centers of China (Guo and Shen 2015a). Furthermore, this area is one of the main agricultural areas in China, producing approximately 10% of the total grain generated in China (Pan et al. 2017). However, according to international standards, the HRB is extremely short of water; the water resources per capita are only 198.6 m^3^ (Tan et al. 2022). Evapotranspiration (ET) is a key component in the water, energy, and carbon cycles (Liu et al. 2021; Pascolini‐Campbell et al. 2021). With regard to the water cycle, ET returns ~60% of precipitation to the atmosphere; this value is even higher in water‐deficient areas (Zeng et al. 2012; Chu et al. 2017, 2019; Lian et al. 2018). At the same time, Latent heat (LE) affects energy partitioning and then impacts the near‐surface environmental factors, such as humidity and temperature (Yang et al. 2019). Therefore, accurate information on ET distributions and impact factor analysis is necessary for water resource management, agricultural development and water cycle and climate change studies (Chen et al. 2022).

Currently, there are a number of studies on the relationships between ET and climate change in the HRB. ET is influenced by several climatic factors, such as radiation, air temperature, humidity, and wind speed, etc. (Tabari and Hosseinzadeh Talaee 2014; Chu et al. 2017; Fisher et al. 2017). Xing et al. (2014) found that the dominant climatic factors for ET differed in different regions of the HRB. The ET in the mountainous regions was lower than in the plain regions, mostly because of lower air temperatures in the former and the influence of irrigation (Guo and Shen 2015a; Li, Chu, et al. 2020). The main controlling factors of ET in the HRB vary with the seasons (Li et al. 2013). Previous studies mainly focused on ET, and there are few studies on the relationships between ET components and climate variables.

The effect of human activity on ET changes is more complicated because the relationships and mechanisms are not sufficiently clear (Wang et al. 2021). Many ET modeling methods do not consider the impacts of human activities such as urbanization and agricultural changes (Wan et al. 2015). Several studies have focused on analyzing the influence of human activities on ET variations in China (Li, Chu, et al. 2020). For example, Lv et al. (2019) researched the influence of human‐related indicators on ET management in a watershed of the Loess Plateau in China. The results indicated that human activities contributed approximately 90% to the ET variations (Lv et al. 2019). The response of ET to land use and land cover change (LUCC) in the Huai River Basin in China was studied by Li et al. (2021), who found that the average annual ET was closely related to land use type. Furthermore, the effects of human activity on ET in future climate change scenarios were investigated in the Heihe agricultural region in China (Zou et al. 2020). The Beijing‐Tianjin Sand Source Control Project started in 2020 significantly changed the LUCC in HRB (Yue et al. 2024). It is important to study the effect of changes in vegetation cover on ET in HRB.

At present, little is understood about changes in ET with both climate change and vegetation change in the HRB. There are also few studies on the attribution analysis of changes in ET components. Studying the spatiotemporal variation of ET, the effects of climate and vegetation parameters on ET variation, and the feedback mechanism between ET and climate change in the HRB in recent years are issues that urgently need to be resolved. Therefore, the objectives of this study are (1) using a satellite‐based model to estimate daily ET and its components (i.e., canopy transpiration, soil evaporation, and interception evaporation) in the HRB with a spatial resolution of 500 m; (2) evaluating the spatial and temporal variations of climate and vegetation variables, and ET and its components in the HRB based on trend analysis; (3) investigating the effects of climate and vegetation factors on ET and its components in the HRB based on correlation analysis and attribution analysis methods.

## 2 Materials and Methods

### 2.1 Study Area

**FIGURE 1:**
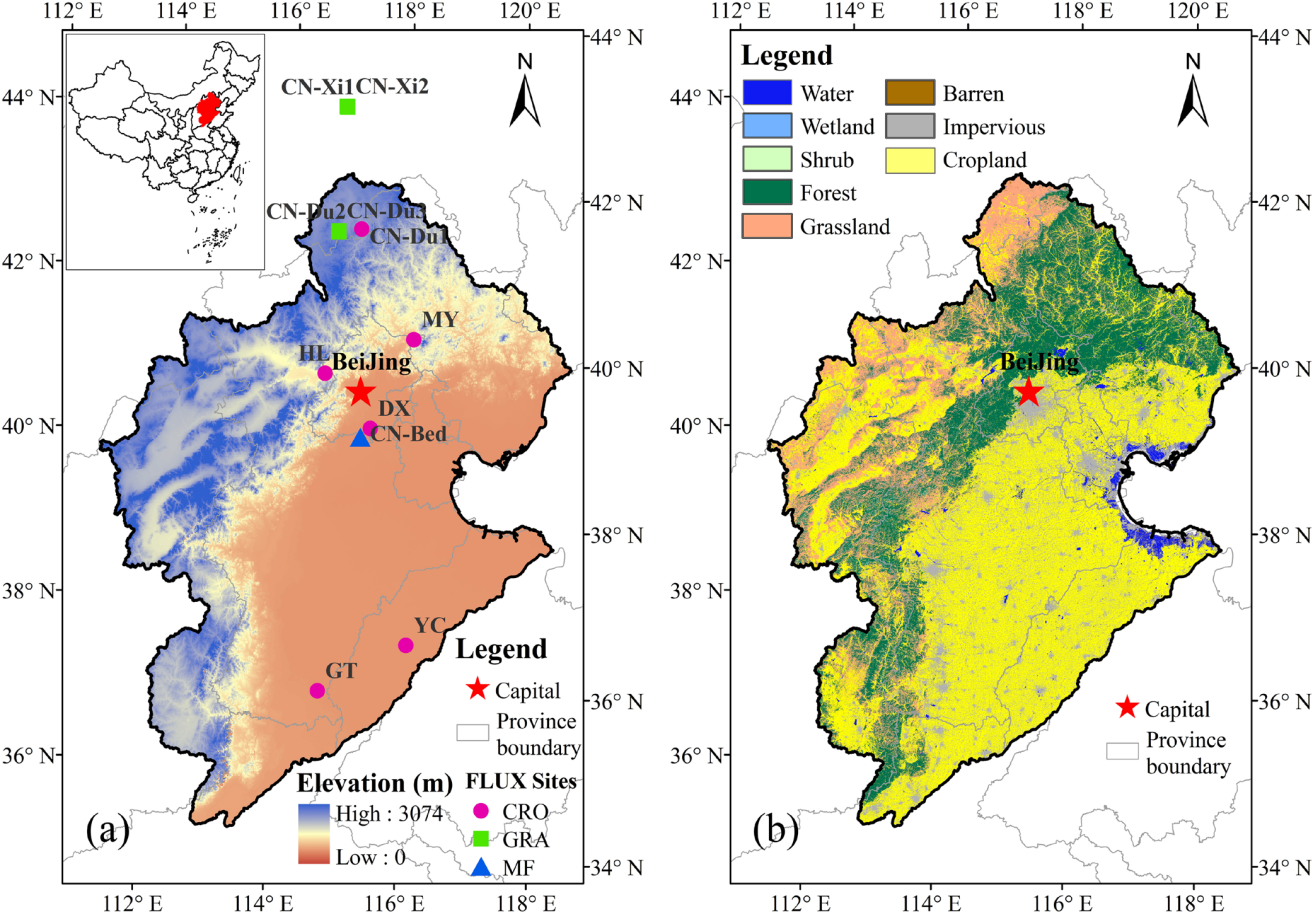
(a) Geographic information of the Haihe River Basin (HRB) and the eddy covariance flux sites used in this study (CRO: Cropland; GRA: Grassland; MF: Mixed forest). (b) The land cover types in the HRB.

The HRB is the largest watershed in North China, lying between 112° E–120° E and 35° N–43° N, and covering a total area of ~320,000 km^2^. The HRB is located in a semi‐moist/semi‐arid region, with a temperate monsoon climate (Jia et al. 2012). There are 448 meteorological stations in or around the HRB. According to the method of Salam et al. (2020), we analyzed the climatic information of the HRB. The long‐term daily average net radiation, air temperature, minimum temperature, maximum temperature, relative humidity, and wind speed across the HRB are 7.25 MJ/m^2^/day, 9.89°C, 4.58°C, 16.21°C, 59.26%, and 2.33 m/s respectively. The annual average total precipitation is 522.06 mm/year. The major crops are wheat and maize, with the yields contributing about 30% and 20% to the total production levels in China, respectively (Pan et al. 2017). Water security is becoming one of the major concerns for this basin (Ma et al. 2023). The land‐cover types in this basin include grasslands, croplands, forests, water bodies, wetlands, built‐up land, and barren land (Yang and Huang 2021). The location and the land‐cover type distribution are shown in Figure 1.

### 2.2 Data

#### 2.2.1 In Situ Measurements

Flux tower‐based measurements were used to validate the ET model. The 11 eddy covariance (EC) sites in or around the HRB are shown in Figure 1 and Table 1. The vegetation types are forest, croplands, and grasslands. The observations include model forcing parameters such as air temperature (Tm), relative humidity (Rh), vapor pressure deficit (VPD), and net radiation (Rn).

**TABLE 1:** Information on the eddy covariance (EC) flux towers.

Site name	Location	Time period	Vegetation type	Sources
CN‐Bed	39.53° N,116.25° E	2005–2006	Mixed forest	LaThuileFlux
CN‐Du1	42.05° N,116.67° E	2006–2006	Cropland	LaThuileFlux
CN‐Du2	42.05° N,116.28° E	2006–2008	Grassland	FLUXNET2015
CN‐Du3	42.06° N,116.28° E	2009–2010	Grassland	FLUXNET2015
CN‐Xi1	43.55° N,116.68° E	2006	Grassland	LaThuileFlux
CN‐Xi2	43.55° N,116.67° E	2006	Grassland	LaThuileFlux
DX2	39.62° N,116.43° E	2008–2010	Cropland	TPDC
GT	36.52° N,115.13° E	2008–2010	Cropland	TPDC
HL	40.35° N,115.79° E	2013–2017	Cropland	TPDC
MY	40.63° N,117.32° E	2008–2010	Cropland	TPDC
YC	36.95° N,116.60° E	2003–2010	Cropland	ChinaFlux

Abbreviation: TPDC, The National Tibetan Plateau Data Center.

The vegetation indices include leaf area index (LAI) and NDVI data for each site, which were extracted from the Moderate Resolution Imaging Spectroradiometer (MODIS). The details on MODIS LAI and NDVI such as resolution were listed in Table 2.

**TABLE 2:** Information of remote sensing datasets.

Dataset	Variables	Spatial resolution (m)	Temporal resolution	Year span
MOD13Q1	NDVI	250	16‐day	2000–2020
MOD15A2	LAI/f_PAR_	500	8‐day	2000–2020
MCD12Q1	Land cover	500	Yearly	2000–2020
SRTMv4.1	DEM	90	Yearly	2008

Abbreviations: DEM, Digital Elevation Model; LAI, Leaf Area Index; NDVI, Normalized Difference Vegetation Index.

#### 2.2.2 Reanalysis Data and Meteorological Data

Reanalysis of net radiation (Rn) data acquired from the global land data assimilation system version 2 (GLDAS 2.1) product (Rodell et al. 2004). The bilinear interpolation was used to resample the initial resolution of 0.25° to 500 m. The bilinear interpolation method provided a smooth transition for the regional parameters by minimizing the footprint effect. It is one of the most widely used interpolation methods (Li, Zhang, et al. 2020).

The meteorological data at the regional scale used in this study, including air temperature (Tm), relative humidity (Rh), wind speed (Ws) and precipitation (Pre), were interpolated from 448 meteorological stations in or around the HRB. Vapor pressure deficit (VPD) was calculated from the Tm and Rh (Chen et al. 2022). These meteorological station data were obtained on a daily scale from the National Meteorological Information Center at the Chinese Meteorological Administration (https://data.cma.cn/en). The interpolation was conducted by the ANUSPLIN software (Hutchinson 2006).

#### 2.2.3 Remote Sensing Data

The variables from remotely sensed datasets used in this study include NDVI, LAI, the fraction of photosynthetically active radiation (*f*_PAR_), land‐cover type, and a digital elevation model (DEM). The vegetation properties were derived by LAI and NDVI from the MOD15A2 and MOD13Q1 datasets in Collection 6 of MODIS. Meanwhile, the land‐cover type product (MCD12Q1) was also from MODIS with 500‐m spatial resolution. These MODIS products can be accessed from the Level‐1 and Atmosphere Archive & Distribution System Distributed Active Archive Center (LAADS DAAC) (https://ladsweb.modaps.eosdis.nasa.gov/). The SRTMv4.1 DEM dataset is available at the website of https://srtm.csi.cgiar.org/.

#### 2.2.4 Quality Control of the Datasets

To ensure the credibility and suitability of the flux tower‐based measurements, quality control (QC) was carried out using quality assurance (QA) identification of the EC measurements. Meanwhile, the energy balance closure adjustment method was assessed by following the method of Jung et al. (2010). For the NDVI and LAI datasets, the quality was controlled by the QC flags provided by the MODIS products. The QC documents can be used to remove the influence of errors made by cloud shadows and clouds, atmospheric attenuation and other error sources (Liu et al. 2022). Linear interpolation was used to generate the daily NDVI and LAI values. The quality control for the meteorological datasets used in this study was done by the National Meteorological Information Center at the Chinese Meteorological Administration. The availability rate of each meteorological element data is generally above 99%, and the accuracy rate of data is close to 100% (https://data.cma.cn/en). The quality control of the interpolated climatic datasets primarily considered the range of the reasonable values. For example, the Rh should be between 0% and 100%, and the Pre should be positive (Salam et al. 2020).

### 2.3 Methods

#### 2.3.1 ET Model

For the full study of ET and its components, one of the most widely used ET models, namely Priestley‐Taylor Jet Propulsion Laboratory (PT‐JPL), was chosen for this study. This algorithm was proposed by Fisher et al. (2008). The total ET was divided into three components, namely canopy transpiration (ET_c_), soil evaporation (ET_s_), and interception evaporation (ET_i_). Each part was downscaled from the potential evapotranspiration (PET) estimated by the Priestley‐Taylor (PT) equation to actual ET. During this downscaling process, the dynamic coefficients calculated by the atmospheric moisture and vegetation indices were utilized. The specific calculation equations are as follows:

(1) $$\mathrm{ET}={\mathrm{ET}}_{c}+{\mathrm{ET}}_{s}+{\mathrm{ET}}_{i},$$

(2) $${\mathrm{ET}}_{c}=\left(1-f_{\mathrm{wet}}\right)f_{g}f_{T}f_{M}\alpha \frac{\Delta }{\Delta +\gamma }R_{\mathrm{nc}},$$

(3) $${\mathrm{ET}}_{s}=\left(f_{\mathrm{wet}}+f_{\mathrm{SM}}\left(1-f_{\mathrm{wet}}\right)\right)\alpha \frac{\Delta }{\Delta +\gamma }\left(R_{\mathrm{nc}}-G\right),$$

(4) $${\mathrm{ET}}_{i}=f_{\mathrm{wet}}\alpha \frac{\Delta }{\Delta +\gamma }R_{\mathrm{nc}},$$

where *G* is the ground heat flux, *f*_wet_ is the relative surface wetness, *f*_g_ is the green canopy fraction, *f*_T_ is the plant temperature constraint, *f*_M_ is the plant moisture constraint, *f*_SM_ is the soil moisture constraint, *α* is the PT coefficient, which is equal to 1.26, Δ is the slope of the saturation‐to‐vapor pressure curve, *γ* is the psychrometric constant (~0.066 kPa/°C), *R*_nc_ is the net radiation allocated to the canopy, and *R*_ns_ is the proportion of the net radiation reaching the soil surface. The Beer–Lambert law (Lambert 1760; Beer 1852) was used to partition the net radiation between the canopy and soil surface. The extinction coefficient *k*_Rn_ in this method was equal to 0.6 (Impens and Lemeur 1969). The detailed computing method of net radiation partitioning is as follows:

(5) $$R_{\mathrm{ns}}=R_{n}\exp\left(-k_{\mathrm{Rn}}\mathrm{LAI}\right),$$

(6) $$R_{\mathrm{nc}}=R_{n}-R_{\mathrm{ns}}.$$

In the PT‐JPL, LAI was the total green and non‐green leaf area index, which was estimated as follows:

(7) $$\mathrm{LAI}=\left(-\ln\left(1-f_{\text{IPAR}}/k_{\mathrm{PAR}}\right)\right),$$

(8) $$f_{\text{IPAR}}=m_{2}\text{NDVI}+b_{2},$$

where *f*_IPAR_ is the fraction of PAR intercepted by total vegetation cover, *k*_PAR_ = 0.5, *m*_2_ = 1.0, and *b*_2_ = −0.05 (Fisher et al. 2008).

#### 2.3.2 Attribution Analysis Method

The PT‐JPL ET model was driven by Rn, Ta, Rh and NDVI. Two types of experimental simulation were performed to evaluate the relative contribution of the major environmental variables to variation in ET and each component, including net radiation, temperature, water condition (relative humidity, Rh) and vegetation conditions (as normalized difference vegetation index, NDVI). The first simulation experiment (*S*_ALL_) was a normal model run, and all environmental drivers were set to vary with time. The second type of simulation experiment (*S*_CLI0_ and *S*_NDVI0_) was a parameter‐controlled experiment. The experimental setup was to maintain one constant at an initial baseline level and to change the other driving factors over time (Yuan et al. 2019; Zheng et al. 2020).

The ET relationships with NDVI and climate variables were estimated through the differences between the simulation results of the first and second types of experiments:

(9) $$\mathrm{\Delta ET}{\left(S_{\mathrm{ALL}}-S_{\text{NDVI}0}\right)}_{i}=\beta_{\text{NDVI}}\times \Delta \text{NDVI}{\left(S_{\mathrm{ALL}}-S_{\text{NDVI}}\right)}_{i}+\varepsilon ,$$

(10) $$\begin{array}{ll} \mathrm{\Delta ET}{\left(S_{\mathrm{ALL}}-S_{\mathrm{CLI}0}\right)}_{i}\;=\; & \beta_{\mathrm{Tm}}\times \mathrm{\Delta Tm}{\left(S_{\mathrm{ALL}}-S_{\mathrm{CLI}0}\right)}_{i}\\ & +\beta_{\mathrm{Rh}}\times \mathrm{\Delta Rh}{\left(S_{\mathrm{ALL}}-S_{\mathrm{CLI}0}\right)}_{i}\\ & +\beta_{\mathrm{Rn}}\times \mathrm{\Delta Rn}{\left(S_{\mathrm{ALL}}-S_{\mathrm{CLI}0}\right)}_{i}+\varepsilon , \end{array}$$

where ΔET denotes the differences in the ET simulations. ΔNDVI, ΔTm, ΔRh, and ΔRn denote the differences in the NDVI, Tm, Rh, and Rn time series between the two types of experiments. *ε* denotes the stochastic error term and was estimated using the maximum likelihood analysis.

#### 2.3.3 Turning Point Analysis

The potential turning point (TP) was detected by a piecewise linear regression method (Yuan et al. 2019) as follows:

(11) $$y=\left\{\begin{array}{l} \beta_{0}+\beta_{1}t+\varepsilon ,\left(t\leq a\right)\\ \beta_{0}+\beta_{1}t+\beta_{2}\left(t-a\right)+\varepsilon ,\left(t>a\right) \end{array}\right.,$$

where *y* is the parameter being analyzed, such as Rn, Ta, Rh or NDVI; *t* indicates the year; *a* is the estimated TP, which means the time when the trend change occurred; *β*_0_, *β*_1_, and *β*_2_ are the regression coefficients; and ε is the residual.

#### 2.3.4 Statistical Analysis

The correlation coefficient (*R*), the root‐mean‐square error (RMSE), bias error and Nash‐Sutcliffe efficiency coefficient (NSE) were used to evaluate the model performance and the relationships between parameters. NSE indicated the magnitude of the residual variance, and the optimal value is 1. If NSE = 0, the model simulations are close to the average level of the observations. NSE < 0 means the performance of the model is unacceptable. These statistical indexes were calculated as:

(12) $$\sigma_{1}={\left[\frac{1}{N}\sum_{i=1}^{N}{\left(P_{1,i}-\bar{P_{1}}\right)}^{2}\right]}^{\frac{1}{2}},$$

(13) $$\sigma_{2}={\left[\frac{1}{N}\sum_{i=1}^{N}{\left(P_{2,i}-\bar{P_{2}}\right)}^{2}\right]}^{\frac{1}{2}},$$

(14) $$R=\frac{\mathrm{COV}\left(P_{1},P_{2}\right)}{\sigma_{1}\sigma_{2}}=\frac{\sum_{i=1}^{N}\left(P_{1,i}-\bar{P_{1}}\right)\left(P_{2,i}-\bar{P_{2}}\right)}{\sqrt{\sum_{i=1}^{N}{\left(P_{1,i}-\bar{P_{1}}\right)}^{2}\sum_{i=1}^{N}{\left(P_{2,i}-\bar{P_{2}}\right)}^{2}}},$$

(15) $$\text{RMSE}={\left[\frac{1}{N}\sum_{i=1}^{N}{\left(ET_{s,i}-ET_{o,i}\right)}^{2}\right]}^{\frac{1}{2}},$$

(16) $$\text{bias}={\bar{\mathrm{ET}}}_{s}-\bar{{\mathrm{ET}}_{o}},$$

(17) $$\mathrm{NSE}=1-\frac{\sum_{i=1}^{N}{\left(ET_{s,i}-ET_{o,i}\right)}^{2}}{\sum_{i=1}^{N}{\left(ET_{o,i}-\bar{ET_{o}}\right)}^{2}},$$

where ET_o_ is the ET observations, $\bar{{\mathrm{ET}}_{o}}$ is the mean of the ET observations, ET_e_ is the ET estimations, $\bar{{\mathrm{ET}}_{e}}$ is the mean of the ET estimations. *P*_1_ and *P*_2_ are two parameters which need to be calculated the correlation coefficient. *σ*_1_ and *σ*_2_ are the deviations of *P*_1_ and *P*_2_, respectively, and COV(*P*_1_, *P*_2_) is the covariance between *P*_1_ and *P*_2_.

## 3 Results

### 3.1 Model Validation

**FIGURE 2:**
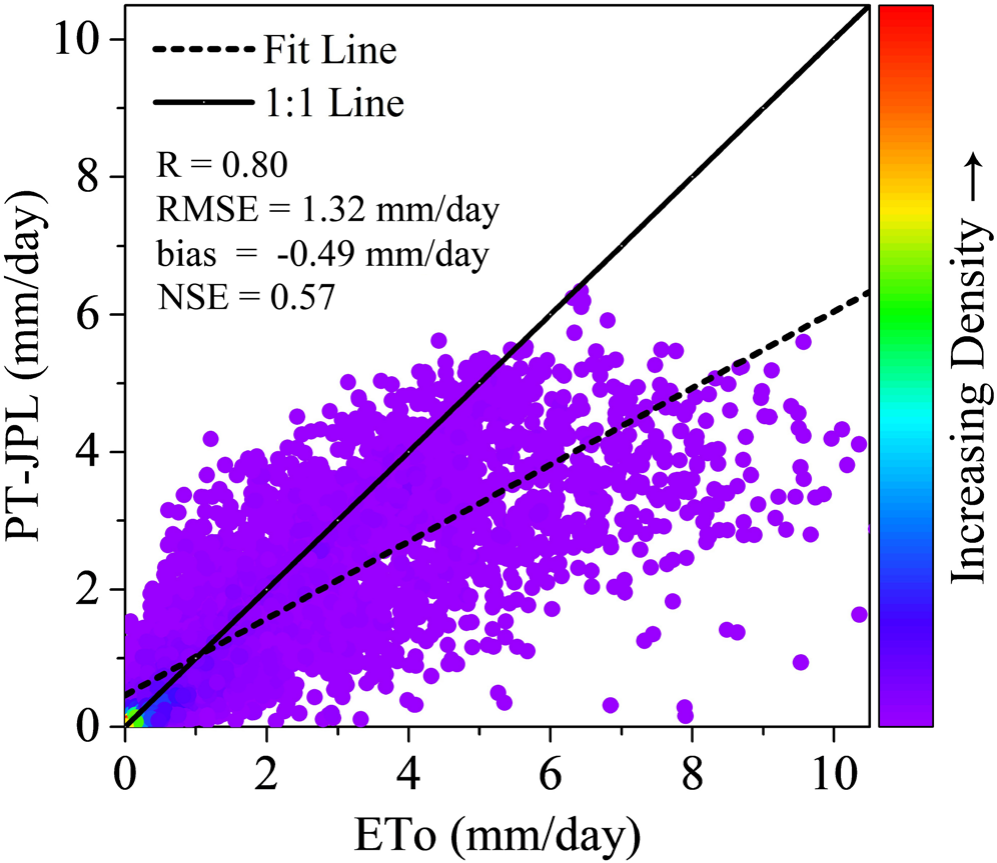
Scatter density plots between the daily PT‐JPL‐simulated ET (PT‐JPL) and EC‐observed ET (ET_o_).

On the daily scale, the ET simulated by the PT‐JPL model showed close agreement with the ET observed by the EC flux tower (Figure 2). The *R* value, RMSE, and NSE values were 0.8, 1.32 mm/day, and 0.57, respectively. Although the PT‐JPL model underestimated the ET measurements with the bias of −0.49 mm/day, the data points with high density were concentrated around the 1:1 line. The average daily ET of a large number of data points was below 1 mm/day. The observed average daily ET ranged from 0 to 10 mm/day.

### 3.2 Spatial Distributions of ET and Climatic and Vegetation Parameters

**FIGURE 3:**
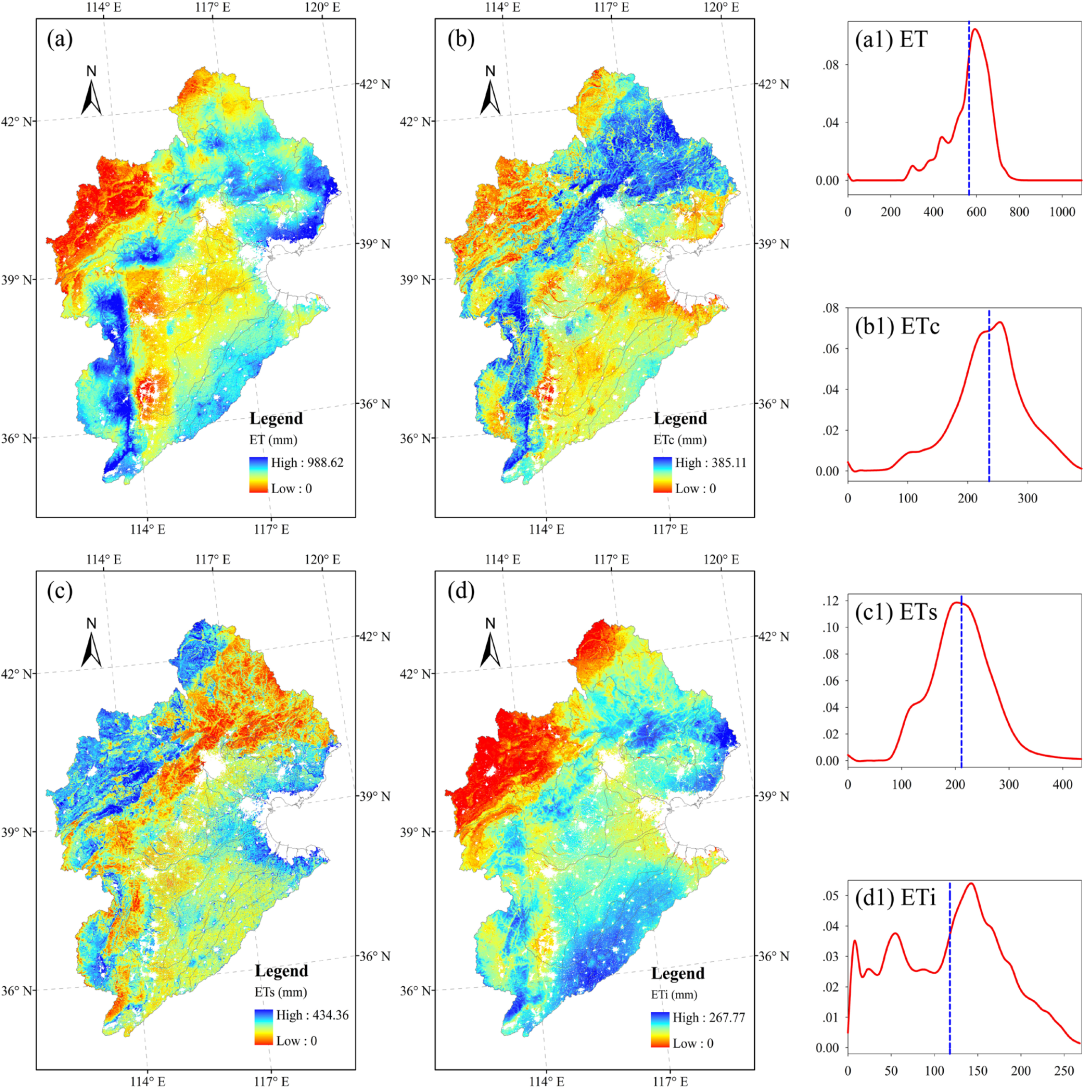
Spatial patterns of (a) ET, (b) ET_c_, (c) ET_s_, and (d) ET_i_. Density plots of (a1) ET, (b1) ETc, (c1) ETs, and (d1) ET_i_. Dashed blue lines refer to the corresponding average values.

The spatial pattern of annual ET and its components (i.e., ET_c_, ET_i_, and ET_s_) from 2000 to 2020 in the HRB is shown in Figure 3. The annual mean ET was found to be higher in the northeast and southwest, where the land was covered by forests (Figure 3a). The lower values were mainly located in the west and southeast, where the land was mainly covered with grassland or crops (Figure 3a). The annual range of ET was 135–989 mm, with a mean value of 582 mm (Figure 3a1). Annual ET and its components exhibited large regional differences. The spatial patterns of ET_c_ were almost consistent with those of ET (Figure 3b). In contrast, the ET_s_ patterns showed roughly opposite distributions to ET and ET_c_, with higher values located in the northwest and lower values located in the northeast and southwest (Figure 3c). Most ET_s_ values were located around 200 mm (Figure 3c1). The spatial pattern of ET_i_ showed regional characteristics different from those of the other two components. Areas with high ET_i_ were mainly distributed in the regions where land was largely covered by forest and summer maize (Figure 3d).

**FIGURE 4:**
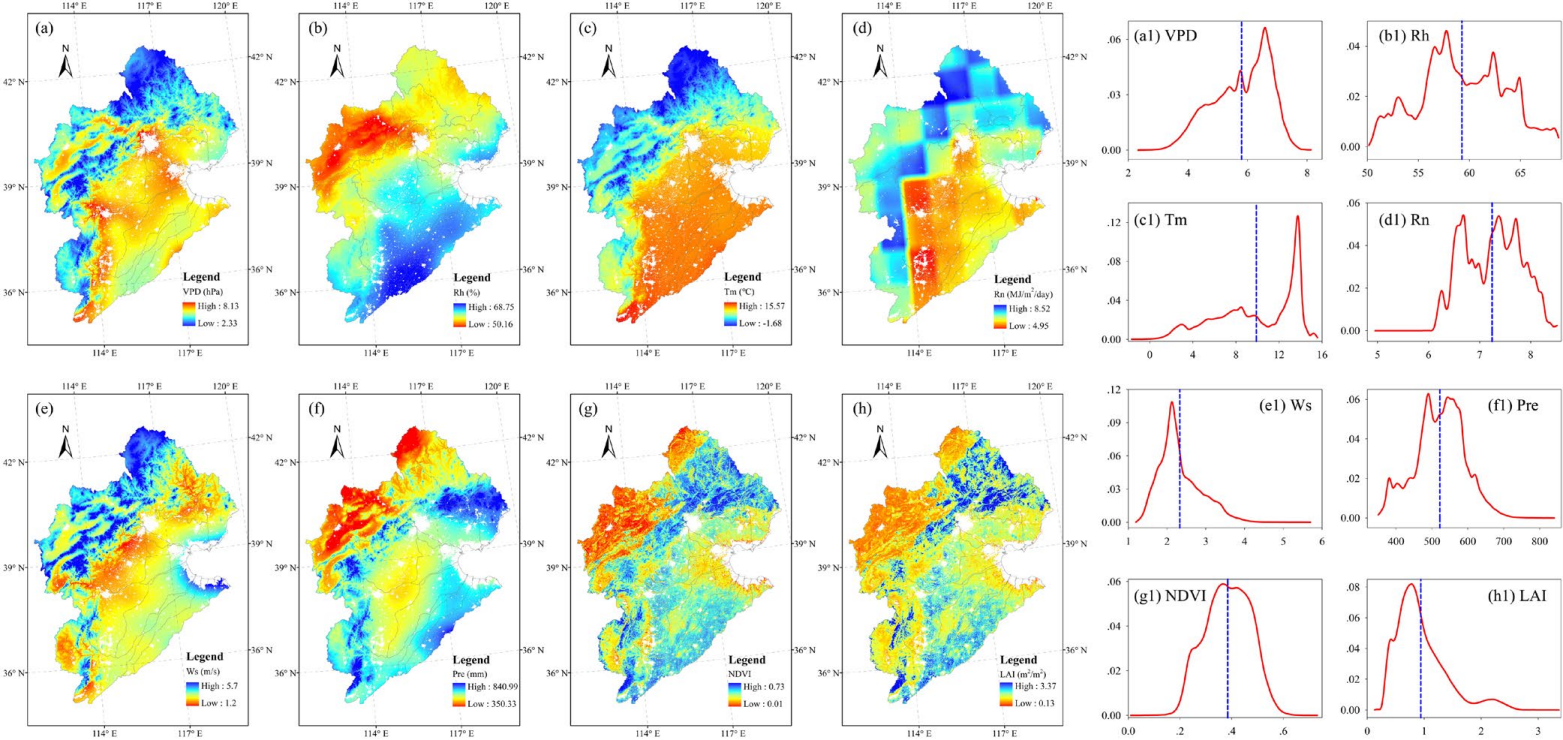
Spatial patterns of (a) VPD, (b) Rh, (c) Tm, (d) Rn, (e) Ws, (f) Pre, (g) NDVI, and (h) LAI. Density plots of (a1) VPD, (b1) Rh, (c1) Tm, (d1) Rn, (e1) Ws, (f1) Pre, (g1) NDVI, and (h1) LAI. Dashed blue lines refer to the average values.

The spatial distributions of climatic variables and LAI are shown in Figure 4. VPD, Tm, Rn and Ws showed similar patterns, with higher values located in the north and west and lower values distributed in the east (Figure 4a–e). On the contrary, Rh showed higher values in the southeast and lower values in the northwest (Figure 4b). NDVI, LAI and Pre had different spatial distribution characteristics compared with other parameters. The lower NDVI and LAI were mainly associated with the grassland areas, whereas the higher values were mainly located in the areas covered by forests and croplands (Figure 4g,h). Regions with high rainfall corresponded to the forest and cropland areas, a distribution similar to the spatial pattern of LAI (Figure 4f).

### 3.3 Spatio‐Temporal Changes of ET and Its Components

**FIGURE 5:**
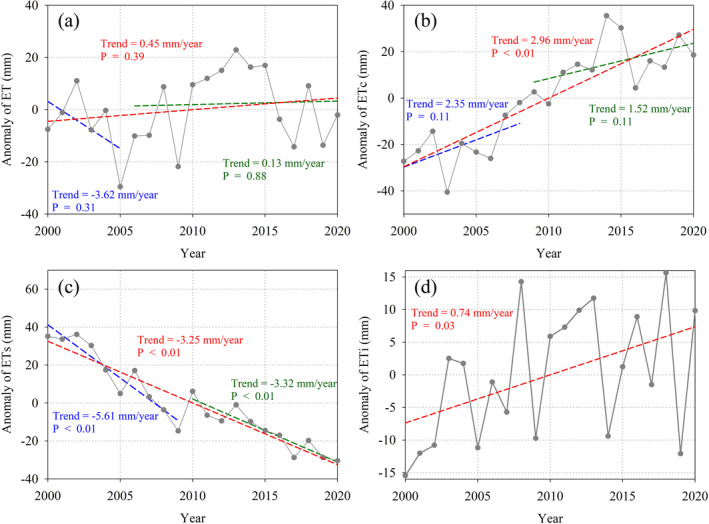
Time series of variations in (a) ET, (b) ET_c_, (c) ET_s_, and (d) ET_i_ from 2000 to 2020.

**FIGURE 6:**
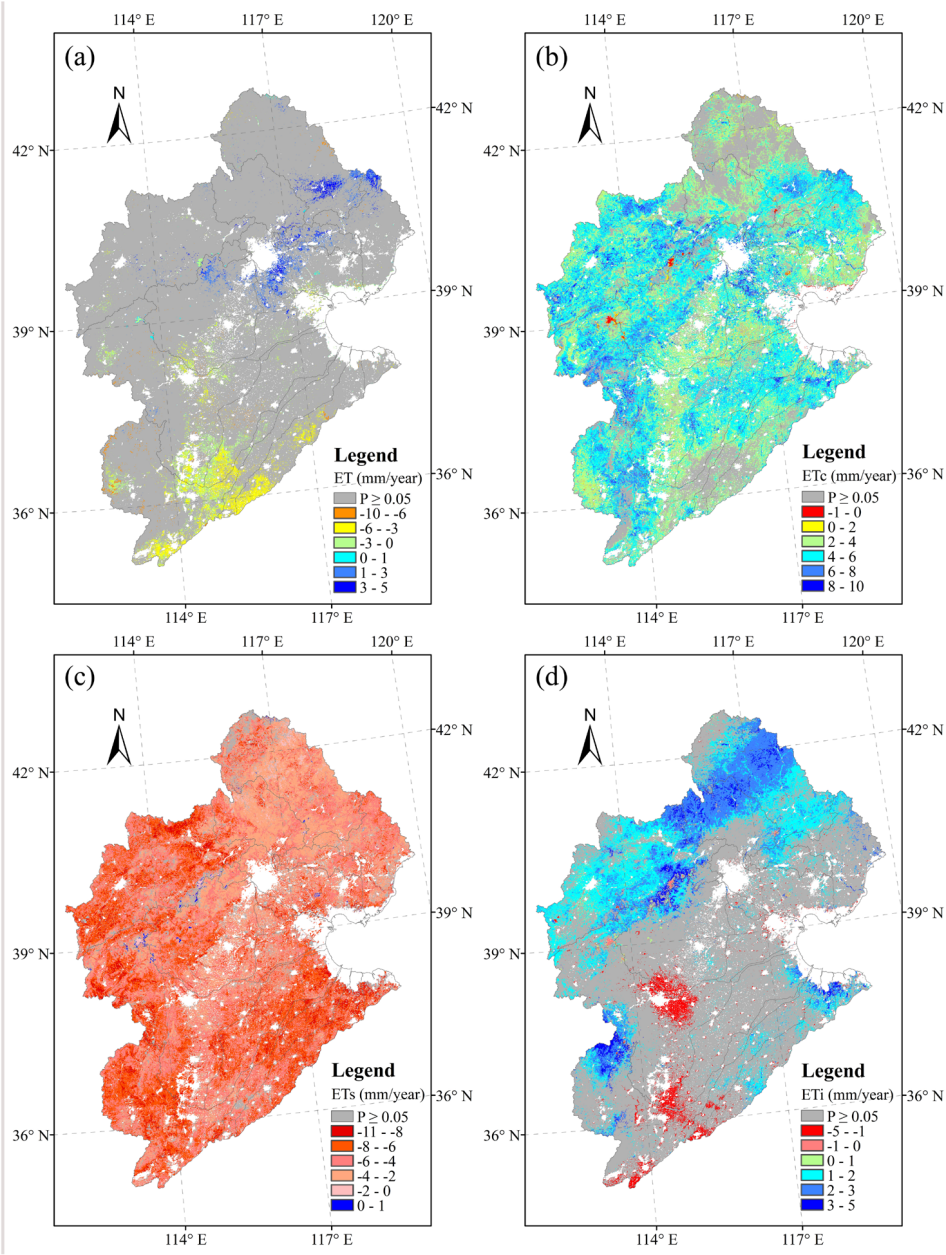
Spatial patterns of trends in (a) ET, (b) ET_c_, (c) ET_s_, and (d) ET_i_ from 2000 to 2020.

The temporal changes of ET and its components are shown in Figure 5. The mean annual total ET from the years 2000 to 2020 showed an increasing trend over time, albeit a non‐significant increase (Figure 5a). The breakpoint appeared in 2006. Before 2006, the ET showed a decreasing trend; after 2006, ET showed an increasing trend (Figure 5a). Regionally, the variation in ET in most areas was not significant (Figure 6a). The areas with significant ET change accounted for only a small proportion of the total area because the changes in ET components canceled one another out. ET_c_ showed a significant increasing tendency over time (Figure 5b), and ET_c_ showed a significant increasing trend across most areas of the HRB (Figure 6b). In contrast, ET_s_ exhibited a significant decreasing trend not only over the period from 2000 to 2020 but also before and after the breakpoint in 2010 (Figure 5c). ET_s_ significantly decreased in response to time over almost the entire HRB region (Figure 6c). ET_i_ showed a significantly increasing trend over time (Figure 5d). ET_i_ on non‐cropland (i.e., forest and grassland) showed a significantly increasing trend over time, whereas ET_i_ on cropland area did not change significantly (Figure 6d).

### 3.4 Changes in Climatic and Vegetation Parameters

**FIGURE 7:**
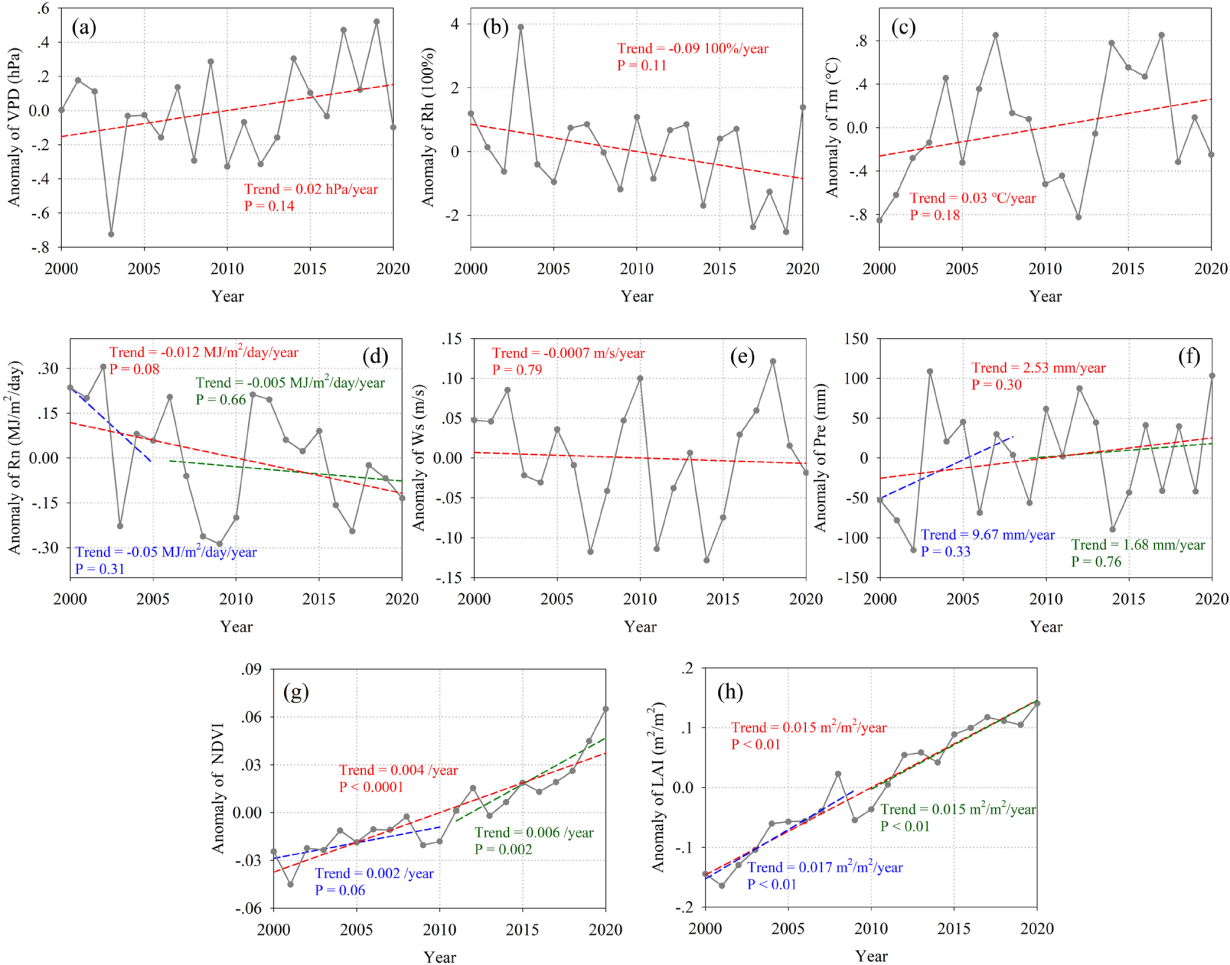
Temporal changes of (a) VPD, (b) Rh, (c) Tm, (d) Rn, (e) Ws, (f) Pre, (g) NDVI, and (h) LAI during 2000 to 2020.

From 2000 to 2020, only NDVI and LAI showed a significant trend toward increasing values with time, with rates of 0.004/year and 0.015 m^2^/m^2^/year, respectively (Figure 7g,h). The parameters VPD, Tm and Pre showed non‐significant increasing trends (Figure 7a,c,f), whereas parameters Rh, Rn and Ws showed non‐significant decreasing trends (Figure 7b,d,e); although the rate of change of Rn did not pass the significance test, the *p* value was close to 0.05 (rate of −0.012 MJ/m^2^/day, *p* = 0.08), so it could merit further study.

**FIGURE 8:**
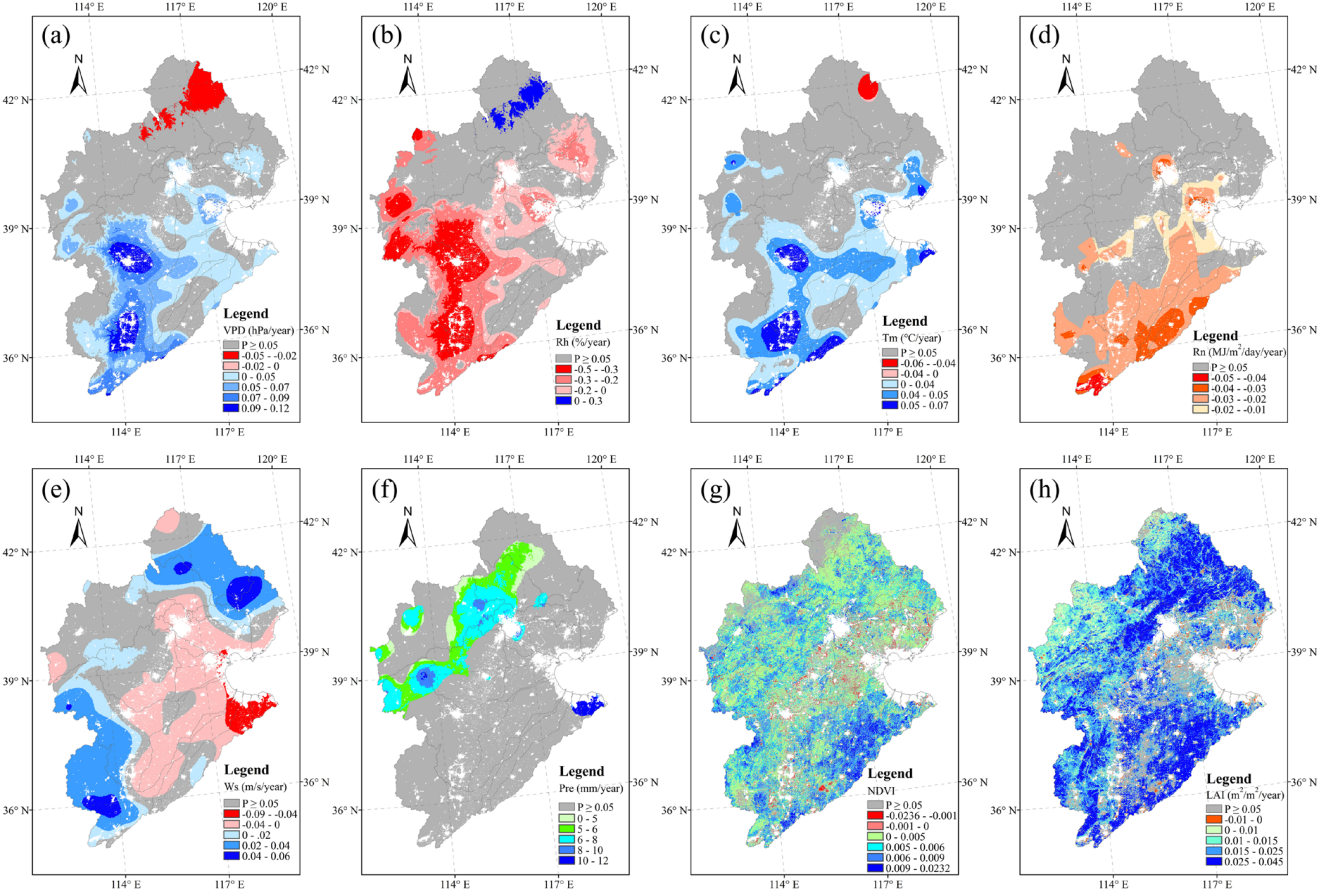
Spatial patterns of trends in (a) VPD, (b) Rh, (c) Tm, (d) Rn, (e) Ws, (f) Pre, (g) NDVI, and (h) LAI from 2000 to 2020.

Figure 8 shows the spatial changes of climatic and vegetation variables during the period from 2000 to 2020. VPD and Tm showed a significant increasing trend in the southern part of the HRB (Figure 8a,c), whereas Rh showed an opposite tendency from that shown by VPD (Figure 8b). At the same time, Rn showed a significant decrease in response to time in the southern part (Figure 8d). Ws showed significant increases in the northeast and southwest parts of the HRB, with significant decreases in the mid‐eastern areas (Figure 8e). For Pre, there was a significant increase in the northern part of the HRB (Figure 8f), whereas, over the study period, NDVI and LAI showed significant increases across almost the entire HRB (Figure 8g,h).

### 3.5 Pearson's Correlation Coefficients Between ET and Climate and Vegetation Variables

**FIGURE 9:**
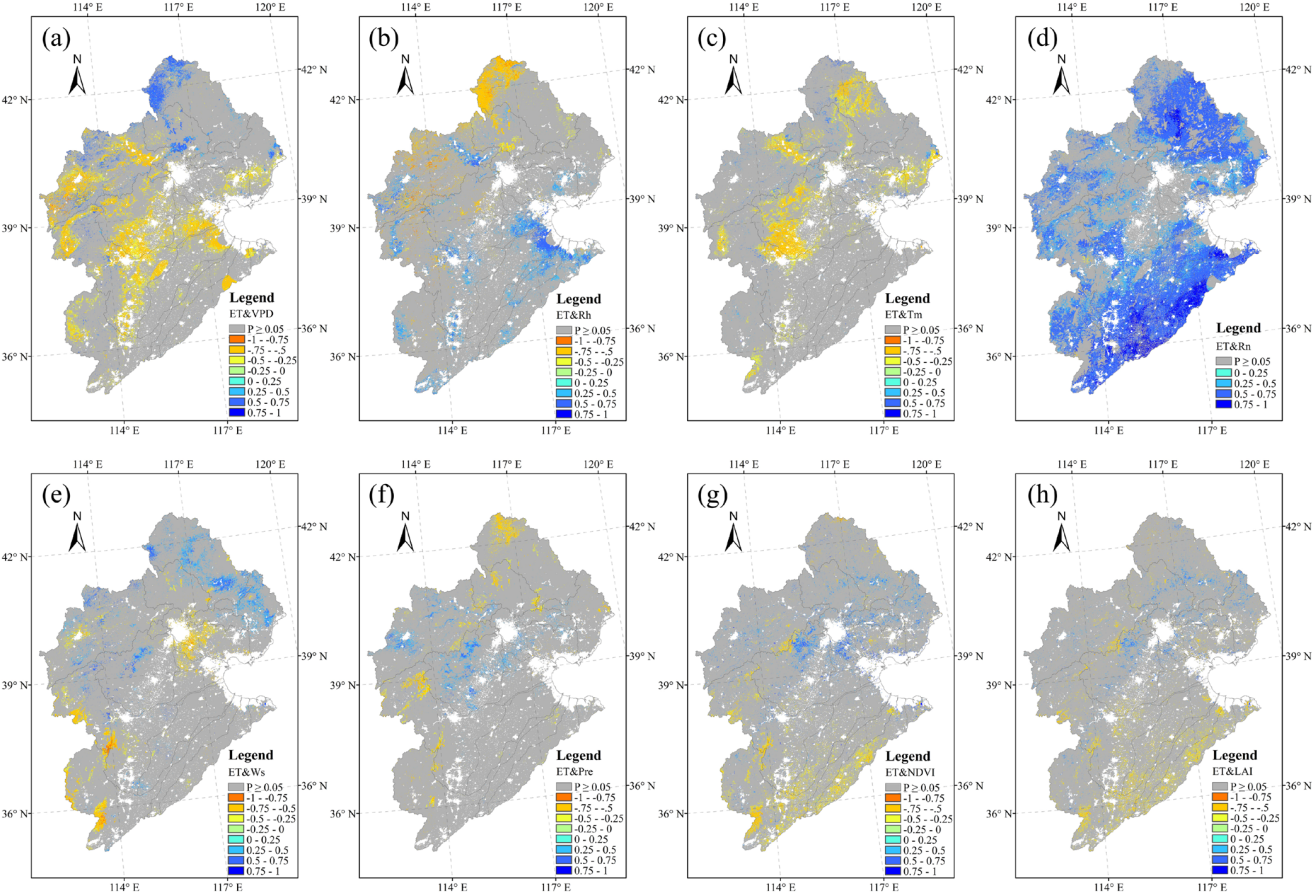
Pearson's correlation coefficients (*R*) between ET and environmental variables. The grids highlighted in gray indicate non‐significant correlations (*p* > 0.05).

ET was affected by variation in climate variables, including solar radiation (Rn), air temperature (Tm), surface moisture (Pre), water conditions (VPD and Rh), and wind speed (Ws), and vegetation variables (NDVI and LAI). Correlation analysis showed that ET and Rn were strongly positively correlated over most regions, particularly in the northern and southern parts of the HRB (Figure 9d). In the central region of the HRB, ET showed significant negative correlations with both VPD and Tm (Figure 9a,c). Ws, NDVI, and LAI showed significant correlations with ET, mainly in the forest areas (Figure 9e,g,h).

**FIGURE 10:**
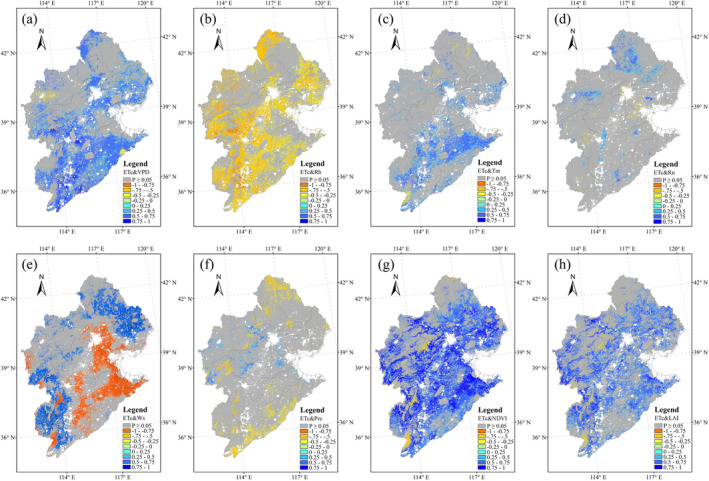
Pearson's correlation coefficients (*R*) between ET_c_ and environmental variables. The grids highlighted in gray indicate insignificant correlations (*p* > 0.05).

**FIGURE 11:**
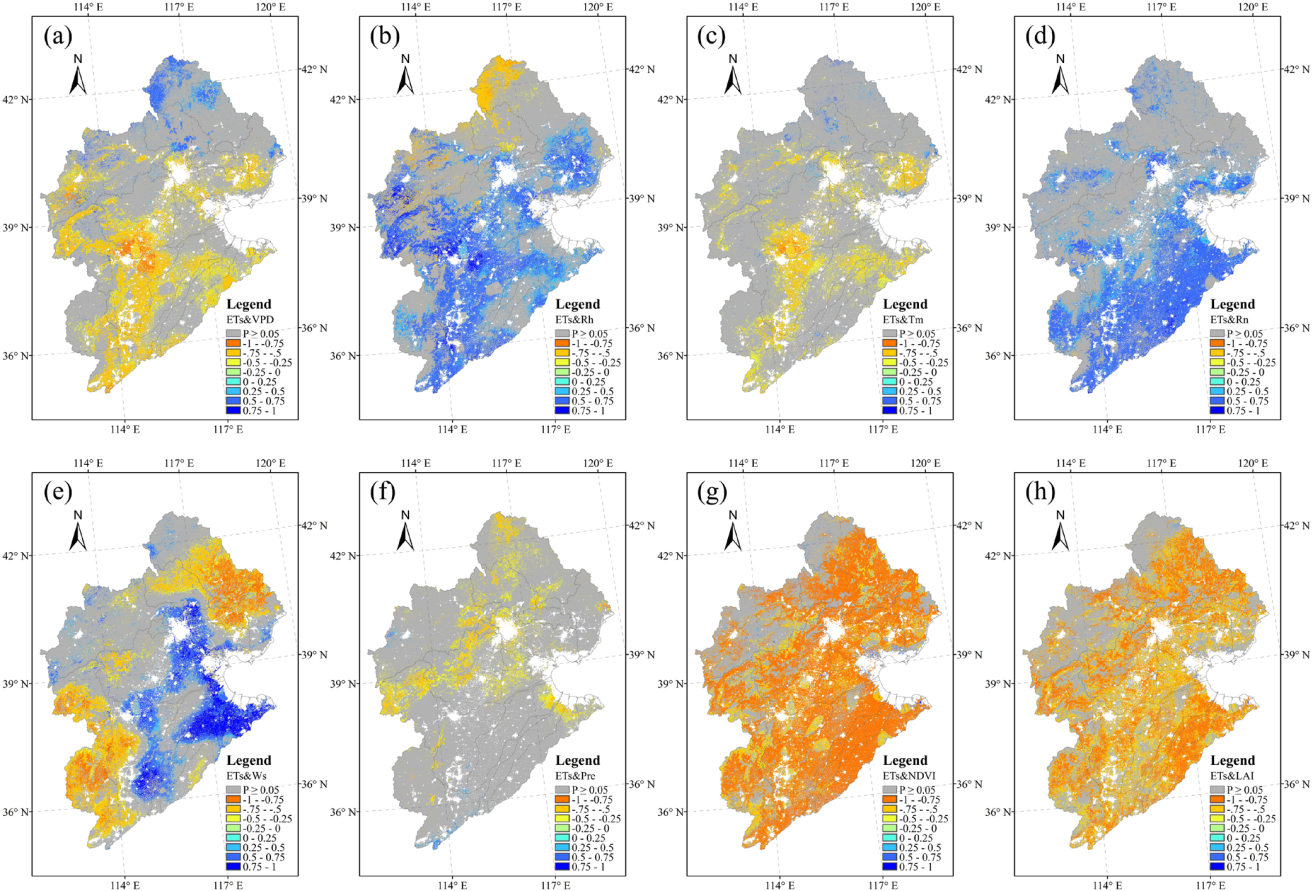
Pearson's correlation coefficients (*R*) between ET_s_ and environmental variables. The grids highlighted in gray indicate non‐significant correlations (*p* > 0.05).

**FIGURE 12:**
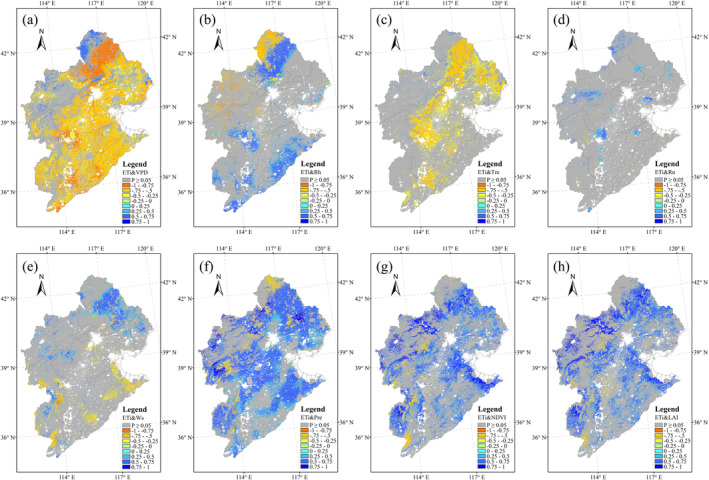
Pearson's correlation coefficients (*R*) between ET_i_ and environmental variables. The grids highlighted in gray indicate non‐significant correlations (*p* > 0.05).

Correlation analyses between different ET components and several climate and vegetation variables showed very different results. The vegetation variables (NDVI and LAI) played an important role in ET_c_ variation (Figure 10g,h), whereas the role of solar radiation was relatively weak (Figure 10d). VPD showed a positive correlation with ET_c_ over the cropland area (Figure 10a), and ET_c_ and air temperature had significant correlations in the cropland area of southern HRB (Figure 10c). Ws influenced ET_c_ in the northern and western regions of the HRB, where the land was mainly covered by grassland (Figure 10e). At the same time, Ws showed a negative correlation with ET_c_ in the cropland area (Figure 10e). With the exception of Rn, the correlation relationships between ET_s_ and its influencing variables showed opposite patterns to the relationships between ET_c_ and the same variables (Figure 11). For example, NDVI and LAI showed negative correlations with ET_s_ over most regions of the HRB (Figure 11g,h), whereas Rn showed significant positive correlations with ET_s_ over most of the cropland area of the HRB (Figure 11d). ET_i_ was mainly controlled by VPD, vegetation variables and Pre over most regions of the HRB (Figure 12). Significant negative correlations between ET_i_ and VPD were exhibited in the HRB (Figure 12a), whereas ET_i_ showed significant positive correlations with both NDVI and Pre (Figure 12f–h).

### 3.6 Attributing ET Variation to Climate and Vegetation Variables

**FIGURE 13:**
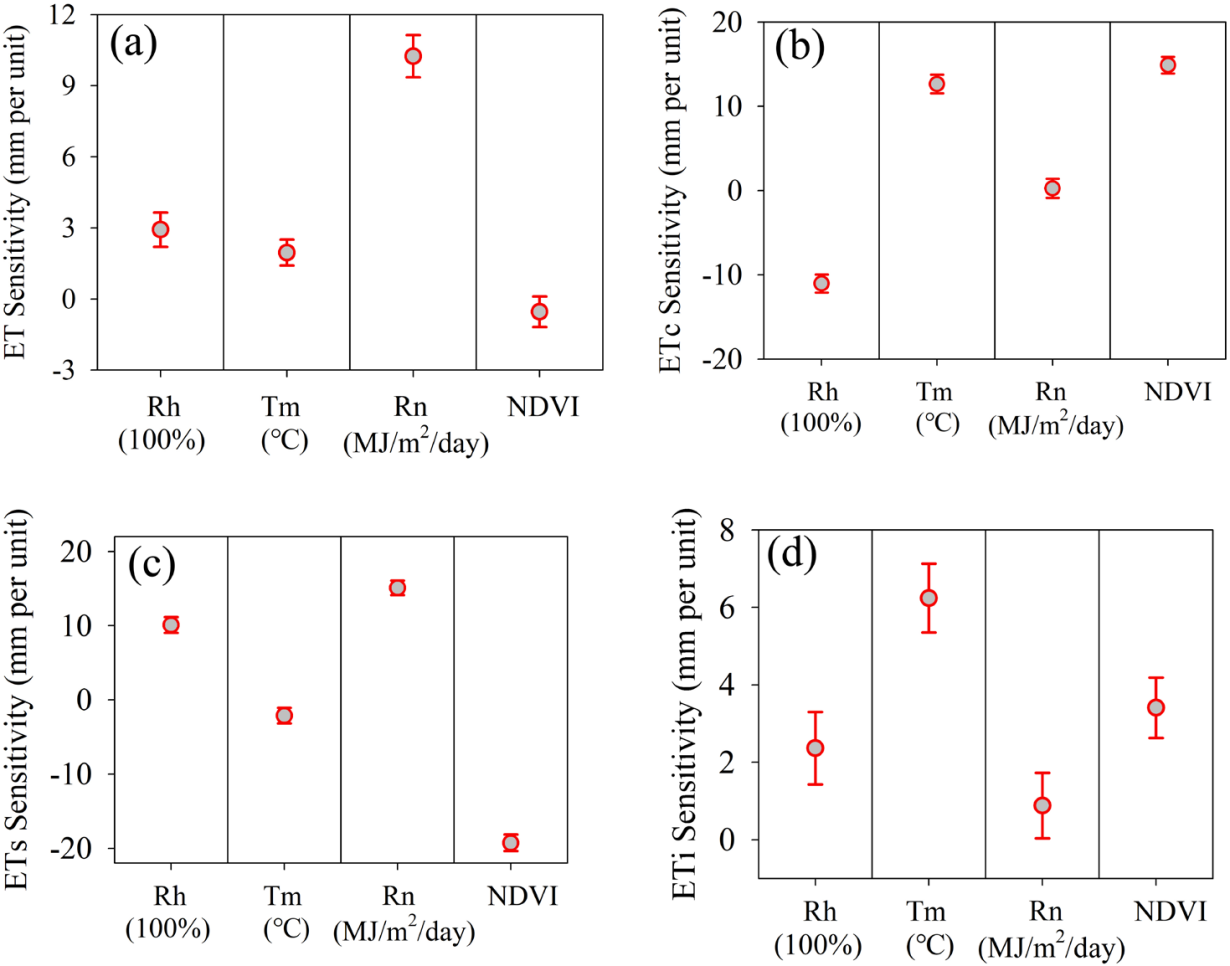
The relationship between ET and its components to climate variables (i.e., Rh, Tm, and Rn) and the vegetation variable NDVI.

To quantify the contributions of several environmental parameters to long‐term changes in ET, ET_c_, ET_s_ and ET_i_, we explored the relationship between ET and its components and climate parameters (i.e., Rh, Tm, and Rn) and the vegetation parameter NDVI (Figure 13). The ET relationship analysis showed that total ET increased by 2.92 ± 0.72 mm per unit increase in Rh, increased by 1.96 ± 0.55 mm/°C with respect to Tm, increased by 10.24 ± 0.89 mm/MJ/m^2^/day with respect to Rn, and decreased by 0.54 ± 0.64 mm per unit increase in NDVI (Figure 13a).

ET_c_ decreased by 11.03 ± 1.07 mm per unit increase in Rh, increased by 12.65 ± 1.10 mm/°C with respect to Tm, increased by 0.27 ± 1.14 mm/MJ/m^2^/day with respect to Rn and increased by 14.89 ± 0.99 mm per unit increase in NDVI (Figure 13b). ET_s_ increased by 10.10 ± 1.07 mm per unit increase in Rh, decreased by 2.13 ± 1.04 mm/°C with respect to Tm, increased by 15.09 ± 0.99 mm/MJ/m^2^/day with respect to Rn and decreased by 19.28 ± 1.11 mm per unit increase in NDVI (Figure 13c). ET_i_ increased by 2.36 ± 0.94 mm per increase in Rh, increased by 6.24 ± 0.89 mm/°C with respect to Tm, increased by 0.88 ± 0.84 mm/MJ/m^2^/day with respect to Rn and increased by 3.41 ± 0.78 mm per unit increase in NDVI (Figure 13d).

## 4 Discussion

### 4.1 Comparison of Satellite‐Based ET Estimation With Other Studies

The spatial patterns of ET in this study were broadly similar to those from previous studies. The high ET values are mainly located in the forest belt from the northeast to the southwest of the HRB areas (Gao et al. 2011; Li et al. 2013; Guo et al. 2022). The high ET values of the forest belt mainly contributed to the high transpiration and interception evaporation of the forest canopy (Figures 1b and 3). From 1961 to 2005, the annual mean ET value in the HRB estimated from a distributed hydrological model was 542 mm/year (Sun and Ren 2013), whereas the annual mean ET estimates from 2005 to 2012 from the in situ water budget, GRACE (Gravity Recovery and Climate Experiment), and MODIS were 515.2, 521.7, and 394.5 mm/year, respectively (Pan et al. 2017). In this study, the annual mean ET from 2000 to 2020 was 582 mm/year, which is close to the values estimated from the hydrological model, GRACE and the in situ water budget. This means the PT‐JPL model in this study could simulate reasonable ET data in HRB. In contrast, the estimates of MODIS showed a large deviation in the HRB because of the overestimation of the low values and underestimation of the high values (Guo et al. 2022). This means that we should be especially careful when choosing ET products. Most previous studies concentrate on the total evapotranspiration estimation in HRB (Gao et al. 2011; Li et al. 2013; Sun and Ren 2013; Guo and Shen 2015a, 2015b; Pan et al. 2017; Chen et al. 2022; Guo et al. 2022), we also estimated the values of ET components, which is very important for understanding the water cycles in HRB. Furthermore, the contributions of several environmental parameters to long‐term changes in ET components were analyzed.

**FIGURE 14:**
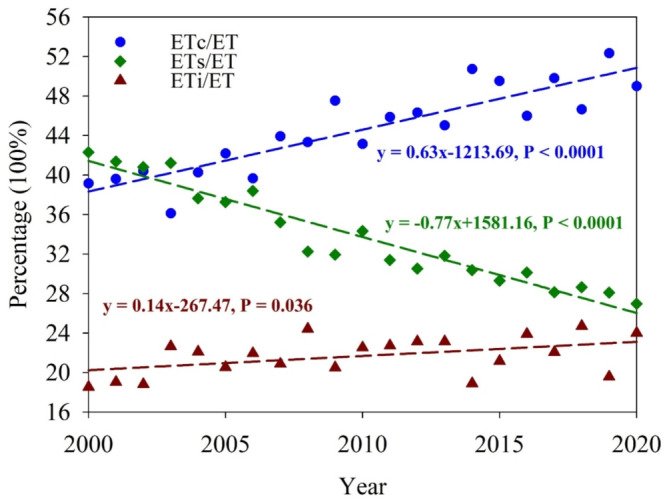
Temporal contributions of the ET key components (ET_c_, ET_s_, and ET_i_) to the total ET from 2000 to 2020.

In this study, the annual mean ET in the HRB showed an increasing trend of 0.45 mm/year from 2000 to 2020. An earlier study reported an annual ET with an increasing trend of 3.7 mm/year from 2000 to 2014 (Yan et al. 2018), compared with the increasing trend over the period 2000 to 2014 in this study, albeit at a lower rate (1.7 mm/year) (Figure 6a). The ET_c_ and ET_i_ in this study also increased significantly, not only from 2000 to 2014 but also over the entire study period (Figure 5), although the significant decrease of ET_s_ over time led to the lower overall rate of ET increase reported in the present study. The opposite changes over time in ET_c_ (increased) and ET_s_ (decreased) reduced the overall rate of ET increase. For the ET components, the ratios of ET_c_ and ET_i_ to ET significantly increased over time, whereas the ratio of ET_s_ to ET showed a significant decrease from 2000 to 2020 (Figure 14). Variations in the climatic and vegetation conditions (Figure 7) led to the change of percentage of the ET components to total ET. Especially, the afforestation and cropland management induced greening of vegetation, which contributes to the increase of the fraction of ET_c_ and ET_i_. This study highlights the importance of illustrating the changes in ET and its components.

### 4.2 Effects of Climate and Vegetation Parameters on ET Variation

The climate parameters were important factors that control ET, including radiation, temperature, and water conditions (Shi et al. 2015; Zhou and Lei 2020; Jerin, Islam, Al Mamun, et al. 2021; Jerin, Islam, Islam, et al. 2021; Li et al. 2021, 2022; Guo et al. 2022). In this study, based on the quantitative analysis method, the roles of radiation, temperature and moisture were identified. The results showed that Rn was the dominant factor in ET variation in the HRB, while Tm and Rh were the two other important factors influencing ET. Rn, Tm, and Rh were the key parameters controlling the ET variations, these results agreed with other studies in the HRB (Guo et al. 2022). We found that the reducing Rn led to the decrease of ET in the southern HRB, which was consistent with the study of Xing et al. (2014). On the other hand, different river basins had different major factors controlling ET. Li et al. (2021) also found that radiation was the leading factor of annual ET variation in a climate transitional zone of eastern China. A study over a humid alpine meadow on the northeastern Qinghai‐Tibetan Plateau also found that net radiation limits ET (Zhang et al. 2017). However, different river basins may have different dominant factors controlling ET. Li, Chu, et al. (2020) indicated that the decreasing Pre and Rh are the main driving factors for the declining ET of the Huaihe River Basin from 2001 to 2014. In the Continental Basin in Northwestern China, water condition was the main factor (Chen et al. 2019). Jerin, Islam, Al Mamun, et al. (2021) and Jerin, Islam, Islam, et al. (2021) reported that the decrease in sunshine duration, and Ws was the main reason for the reduction in reference ET.

Identifying the effects of climate and vegetation parameters on the different ET components is important. Different ET components have different control factors, and the effects of some factors could counteract the effects of other control factors, leading to the false appearance that a particular factor has no influence on total ET. For example, vegetation (i.e., NDVI and LAI) and Tm had large impacts on ET_c_, while Rn and Rh (VPD) played important roles on ET_s_, and Tm and vegetation (NDVI and LAI) were key impact parameters for ET_i_ in HRB. LAI had significant positive correlations with ET_c_ and ET_i_ (Figures 10e and 12e), but had a significant negative correlation with ET_s_ (Figure 11e). This is due to the Beer–Lambert law, which is used to partition net radiation between the soil surface (R_ns_) and the canopy (*R*_nc_) (Lambert 1760; Beer 1852). In this law, a negative correlation relationship exists between *R*_ns_ and LAI. After summing the components ET_c_, ET_s_ and ET_i_, the negative correlation between LAI and ET_s_, and the positive correlations between LAI and both ET_c_ and ET_i_ canceled each other out, leading to no significant correlation between LAI and total ET (Figure 9e). The increase of LAI since 2000 is partially a benefit from the forest protection and afforestation projects and cropland management in HRB (Lei et al. 2014; Cao et al. 2024; Yue et al. 2024). Cheng et al. (2021) showed that LAI was the main factor affecting ET in the Pearl River Basin. The ecological project affects the surface vegetation coverage status, thereby influencing the change rate of ET (Cheng et al. 2021). The results in this study accorded with the biophysical mechanism of each ET component (Yang et al. 2023; Villalobos et al. 2024). Before this study, there had been little detailed research on the factors influencing ET components, which has implications for research in other regions of the world.

### 4.3 Uncertainties and Future Studies

This study quantitatively assessed the effects of climate and vegetation changes on ET and its components, but there are still some uncertainties about the datasets and methods. First, each climate variable is not completely independent and may lead to uncertainty in the results (Li et al. 2022). Second, the vegetation and climate variables are also not independent. The change of vegetation is the result of human activities and climate changes. The direct and indirect effects of climate and vegetation on ET impacts can be quantitatively evaluated in the future by land surface modeling (Li, Chu, et al. 2020). The human activities induced land‐cover change also influence regional climate change (Cao et al. 2020), which can be quantitatively assessed using regional atmospheric models in future studies (Cao et al. 2015). Third, net radiation is an important climate variable affecting ET, but the GLDAS 2.1 dataset with a spatial resolution of 0.25° × 0.25° is slightly coarse (Khan et al. 2018; Wu et al. 2021). Currently, the ERA5‐Land provides the hourly net radiation at a 0.1° spatial resolution (Muñoz‐Sabater et al. 2021). Validation and selection of those net radiation datasets and their effects on ET simulation should be investigated in future studies. Overall, the effects of climate and human activities (e.g., irrigation, fertilization, and afforestation projects) on ET can be assessed through combining the land surface model, atmospheric model and high‐resolution net radiation datasets.

## 5 Conclusions

In this study, the PT‐JPL model was used to estimate the actual ET variation and its components across the HRB during the period 2000 to 2020. The contributions of environmental and vegetation parameters to ET and its components were quantified. The rationality of the ET estimations, the impacts of climate factors and vegetation parameters on ET and its components were discussed. The results indicated that the PT‐JPL model could simulate ET across the HRB reasonably well. The results showed that annual ET increased at a rate of 0.45 mm/year from 2000 to 2020, with the components ET_c_ and ET_i_ increasing significantly over time at rates of 2.96 mm/year (*p* < 0.05) and 0.74 mm/year (*p* < 0.05), respectively. Meanwhile, ET_s_ decreased significantly over time at a rate of 3.25 mm/year (*p* < 0.05). The quantitative relationship studies between ET (and its components) and climate/vegetation parameters showed that net radiation (Rn) was the most important driver of ET variation. For the ET components, vegetation index and temperature had large impacts on transpiration, whereas net radiation and water conditions played important roles in evaporation and temperature and vegetation index were key impact parameters for interception. This study emphasizes the importance of quantifying the contributions of climate change and vegetation to the changes in ET and its components, which assist in water resources management in HRB and other areas of the world. In the future, the impacts of climate and human activities (e.g., irrigation, fertilization, and afforestation projects) on ET can be evaluated by combining the land surface model, atmospheric models, and high‐resolution net radiation datasets.

## Author Contributions

**Yang Chen:** conceptualization (supporting), investigation (lead), methodology (lead), validation (lead), writing – original draft (lead). **Se Chai:** formal analysis (equal), validation (equal), writing – review and editing (equal). **Wenjie Chen:** formal analysis (equal), writing – review and editing (equal). **Jiangzhou Xia:** conceptualization (lead), data curation (lead), methodology (supporting), validation (supporting), writing – original draft (supporting).

## Conflicts of Interest

The authors declare no conflicts of interest.

## Data Availability

The dataset provided in this paper can be obtained at https://zenodo.org/records/13370848 (Chen 2024).
